# Hemolysin EthA is an *Edwardsiella* T3SS effector that induces PANoptosis in macrophages

**DOI:** 10.1186/s13567-026-01814-x

**Published:** 2026-07-11

**Authors:** Pu Yu Tang, Xiu Long Jiang, Xin Yong Zhou, Tian Tian He, Pin Nie, Hai Xia Xie

**Affiliations:** 1 Institute of Hydrobiology, Chinese Academy of Sciences, Wuhan, China; 2 College of Advanced Agricultural Sciences, University of Chinese Academy of Sciences, Beijing, China; 3 State Key Laboratory of Breeding Biotechnology and Sustainable Aquaculture, Institute of Hydrobiology, Chinese Academy of Sciences, Wuhan, China

**Keywords:** Hemolysin, T3SS effector, PANoptosis, *Edwardsiella piscicida*

## Abstract

*Edwardsiella piscicida* is an enteric intracellular bacterial pathogen. It has been reported that this bacterium produces two types of hemolysins: the secreted, pore-forming toxin HlyA and the cell-associated toxin EthA. EthA has been reported to enter epithelial cells by binding to outer membrane vesicles (OMVs) and that once inside the host cells, it releases lipopolysaccharide from the OMVs to induce pyroptosis. We report here the existence of another pathway by which EthA is secreted and delivered into host cells: the type III secretion system (T3SS) of *E. piscicida*. The ΔFur box-P_*ethB*_ strain that highly expresses EthA was constructed by deleting the binding motif of Fur in front of the *ethB-ethA* operon. The ΔFur box-P_*ethB*_ strain induces PANoptosis (a combination of pyroptosis, apoptosis and necroptosis) in a T3SS-dependent manner in murine macrophages. The highly expressed EthA stimulates pyroptosis partially via the NLRP3 inflammasome and concomittantly induces apoptosis and necroptosis, as evidenced by elevated ratios of positive cells in TUNEL (terminal deoxynucleotidyl transferase dUTP nick end labeling) staining, elevated protein levels of cleaved caspase-3 and phosphorylated MLKL. PANoptosis is characterized by cell swelling, membrane rupture and cell blebbing. Additionally, the highly expressed EthA inhibits the phosphorylation of the serine/threonine kinase TAK1 and transcription factor p65, preventing the nuclear translocation of phosphorylated p65 into murine macrophages. Overall, EthA has been identified as a novel T3SS substrate in *E. piscicida*. Its high expression inhibits the phosphorylation of TAK1, thereby increasing PANoptosis and blocking the inflammatory pathway.

**Supplementary Information:**

The online version contains supplementary material available at 10.1186/s13567-026-01814-x.

## Introduction

*Edwardsiella piscicida* PPD130/91 (formerly known as *Edwardsiella tarda* PPD130/91) is a Gram-negative, enteric pathogen that mainly causes hemorrhagic septicemia in fish, but it is also emerging as an infectious agent in humans [1, 2]. This bacterium replicates in *Edwardsiella*-containing vacuoles (ECVs) in primary fish macrophages and in the murine macrophage cell lines J774A.1 and RAW264.7. It also replicates in bone marrow-derived macrophages (BMDMs). This replication depends on an active type III secretion system (T3SS) [3–5].

The T3SS is a multi-component, syringe-like structure that is assembled on the surface of bacteria. T3SS effectors, which are delivered into host cells via the T3SS apparatus, promote bacterial survival and replication [6]. To date, seven T3SS effectors have been identified and characterized in terms of their function in *E. piscicida*, in addition to the 11 novel ones recently reported by Peng et al. [7]. Among these, EseQ promotes invasion by disrupting the epithelial barrier [8]; EseG destabilizes host’s microtubules [9]; EseJ promotes the replication of *E. piscicida *in vivo by suppressing the production of reactive oxygen species and blocking the endocytic trafficking of ECVs to lysosomes, while also inhibiting PANoptosis (a combination of pyroptosis, apoptosis and necroptosis) [10–12]; EseH is a phosphothreonine lyase that suppresses mitogen-activated protein kinase (MAPK) signaling and promotes the degradation of transcription factor p65 [13, 14]; EseK inhibits the phosphorylation of MAPK and promotes bacterial colonization in zebrafish larvae [15]; Trx2 suppresses the apoptosis of macrophages; and YfiD inhibits the activation of host poly (ADP-ribose) polymerase-1 to promote *E. piscicida* infection [16, 17]. Recently, a proteomic approach was employed to compare the secretomes of *E. piscicida* between a T3SS gatekeeper mutant that secrete T3SS injectisome effectors at high levels, and a T3SS mutant that fails to secrete T3SS effectors. It was found that the hemolysin EthA is secreted partially in a T3SS-dependent manner [18].

Two types of hemolysins have been identified in *E. piscicida*: the 34.0-kDa secreted pore-forming toxin HlyA and the 165.3-kDa bacterial cell-associated hemolysin EthA [19–21]. EthA is part of the two-partner secretion (TPS) system. The transporter protein TpsB exports its large cognate passenger protein TpsA (hemolysin). EthA, HpmA, ShlA and ExlA are all members of the TPS hemolysin family [22, 23]. The environmental cue that stimulates ShlAB is iron limitation [24]. Fur is an iron uptake regulator that binds to Fe^2+^, enabling it to recognize the Fur box to exert transcription suppression [25]. The Fe^2+^–Fur complex inhibits transcription of the *shlB-shlA* operon in *Serratia marcescens* and the *eihB-eihA* operon in *Edwardsiella ictaluri* [24, 26]. The same is true for the *ethB-ethA* operon in *E. piscicida*, as there are two Fur-binding motifs in the upstream region of the *ethB* gene [19].

TpsA and TpsB are both translocated to the periplasm via the SecYEG translocon. TpsB then assembles in the outer membrane to form a β-barrel, through which TpsA is exported [27]. EthB is the transporter protein responsible for exporting the hemolysin EthA in *E. piscicida* [28] which is delivered into epithelial cells by binding to outer membrane vesicles (OMVs) [29]. The thermostable direct hemolysin (TDH) in *Vibrio parahaemolyticus* employs a unique dual secretion pathway. The Sec signal peptide-containing precursor is first translocated to the periplasm via the SecYEG translocon and is then cleaved by leader peptidase to generate the mature form of TDH (m-TDH). m-TDH is primarily secreted via the type II secretion system (T2SS). Interestingly, some periplasmic m-TDH molecules are re-imported into the cytosol, where they are recognized and translocated into host cells via T3SS2 [30].

The ExlA toxin of the TPS system in *Pseudomonas aeruginosa* induces pyroptotic death in macrophages [31]. Pyroptosis is a highly pro-inflammatory form of lytic cell death. It is characterized by the formation of an inflammasome, the activation of caspase-1 or caspase-4/11, formation of a GSDM pore and maturation and release of interleukin-1β (IL-1β) [32–34]. In addition to pyroptosis, bacterial infection stimulates two other types of programmed cell death (PCD): apoptosis and necroptosis. There are two pathways through which apoptosis can be initiated: intrinsically, through mitochondrial outer membrane permeabilization (MOMP), or extrinsically, through death receptor activation. The process is then carried out by caspase-3 or caspase-7 [35, 36]. Necroptosis does not involve caspases; rather, it is primarily regulated by receptor-interacting protein kinase 1 (RIPK1) and RIPK3 [37]. The activation of RIPK3 leads to the phosphorylation of the mixed lineage kinase domain-like protein (MLKL), which carries out necroptosis [38]. These three types of PCD are interconnected and can transform into one another, inducing different types of cell death [39]. PANoptosis exhibits key features of pyroptosis, apoptosis, and necroptosis. This form of coordinated cell death is activated by specific stimuli and regulated by the PANoptosome complex [40].

This study demonstrates that the hemolysin EthA is primarily secreted and translocated via the T3SS. The T3SS-translocated EthA protein inhibits transforming growth factor β-activated kinase 1 (TAK1) phosphorylation, thereby stimulating PANoptosis and inhibiting the NF-κB signaling pathway. Furthermore, EthA enhances the fitness of *E. piscicida* in fish.

## Materials and methods

### Bacterial strains and cultivation

Table 1 lists the bacterial strains that were labeled with an hemagglutinin (HA) tag, a FLAG tag or a kanamycin (Km) resistance gene cassette, and the plasmids that were used in this study. For brevity, the insertion of a Km resistance gene cassette is not shown in the text, figures or figure legends when referring to the following strains: ΔFur box-P_*ethB*_, Δ*esaN*ΔFur box-P_*ethB*_, WT *ethA*-2HA, Δ*esaN ethA*-2HA, ΔFur box-P_*ethB*_* ethA*-2HA, Δ*esaN*ΔFur box-P_*ethB*_* ethA*-2HA, WT *ethA*-2HA *eseG*-3FLAG, Δ*esaN ethA*-2HA *eseG*-3FLAG, ΔFur box-P_*ethB*_* ethA*-2HA *eseG*-3FLAG, Δ*esaN*ΔFur box-P_*ethB*_* ethA*-2HA *eseG*-3FLAG, ΔFur box-P_*ethB*_/RFP (red fluorescent reporter protein) and Δ*esaN*ΔFur box-P_*ethB*_/RFP. The *E. piscicida* PPD130/91 strain [41] and its derivative strains were grown in tryptic soy broth (TSB) at 28 ℃. To activate the T3SS, *E. piscicida* strains were grown at 25 °C in Dulbecco’s Modified Eagle Medium (DMEM) (Gibco, Thermo Fisher Scientific, Waltham, MA, USA) in an atmosphere containing 5% CO_2_. Antibiotics were added as required at the following concentrations: 100.0 μg/mL ampicillin (Amp; Sigma-Aldrich, St. Louis, MO, USA), 12.5 μg/mL colistin (Col; Sigma-Aldrich), 50.0 μg/mL kanamycin (Km, Solarbio Science & Technology Co., Beijing, China), 34.0 μg/mL chloramphenicol (Cm; Amresco, Solon, OH, USA) and 50.0 μg/mL gentamicin (Gem; Sigma-Aldrich).

**Table 1:** **Strains and plasmids used in this study**

Strain or plasmid	Description and/or genotype	Reference or source
*Edwardsiella piscicida*		
PPD130/91	wild-type, Km^s^, Col^r^, Amp^s^, LD_50_ = 10^5.0^	[41]
Δ*esaN*	PPD130/91, *esaN* in-frame deletion of aa 1–441	[9]
Δ*eseJ*	PPD130/91, *eseJ* in-frame deletion of aa 1–1359	[10]
ΔFur box-P_*ethB*_::Km	PPD130/91, Fur box sequence replaced with Km^r^	This study
Δ*esaN*ΔFur box-P_*ethB*_::Km	Δ*esaN* with Fur binding box upstream of *ethB* replaced with Km^r^	This study
WT/pACYC-*eseG*::*cyaA*	PPD130/91 with pACYC-*eseG*::*cyaA*	[46]
Δ*esaN*/pACYC-*eseG*::*cyaA*	Δ*esaN* with pACYC-*eseG*::*cyaA*	[46]
WT/pACYC-*ethA*_1-338 aa_::*cyaA*	PPD130/91 with pACYC-*ethA*_1-338 aa_::*cyaA*	This study
Δ*esaN*/pACYC-*ethA*_1-338 aa_::*cyaA*	Δ*esaN* with pACYC-*ethA*_1-338 aa_::*cyaA*	This study
Δ*ethA*	PPD130/91, *ethA* in-frame deletion of aa 1 to 1614	This study
WT *ethA*-2HA::Km	PPD130/91, with chromosomal *ethA* tagging with 2HA, Col^r^, Km^r^	This study
Δ*esaN ethA*-2HA::Km	Δ*esaN* with chromosomal *ethA* tagging with 2HA, Col^r^, Km^r^	This study
ΔFur box-P_*ethB*_* ethA*-2HA::Km	ΔFur box-P_*ethB*_ with chromosomal *ethA* tagging with 2HA, Col^r^, Km^r^	This study
Δ*esaN*ΔFur box-P_*ethB*_* ethA*-2HA::Km	Δ*esaN*ΔFur box-P_*ethB*_ with chromosomal expression of EthA-2HA, Col^r^, Km^r^	This study
WT *ethA*-2HA *eseG*-3FLAG::Km	PPD130/91 with chromosomal *ethA* and *eseG* tagging respectively with 2HA and 3FLAG, Col^r^, Km^r^	This study
Δ*esaN ethA*-2HA *eseG*-3FLAG::Km	Δ*esaN* with chromosomal *ethA* and *eseG* tagging respectively with 2HA and 3FLAG, Col^r^, Km^r^	This study
ΔFur box-P_*ethB*_* ethA*-2HA *eseG*-3FLAG::Km	ΔFur box-P_*ethB*_ with chromosomal *ethA* and *eseG* tagging respectively with 2HA and 3FLAG, Col^r^, Km^r^	This study
Δ*esaN*ΔFur box-P_*ethB*_* ethA*-2HA *eseG*-3FLAG::Km	Δ*esaN*ΔFur box-P_*ethB*_ with chromosomal expression of EthA-2HA and EseG-3Flag, Col^r^, Km^r^	This study
WT/RFP	PPD130/91 transformed with pFPV-*rfp*	[45]
Δ*esaN*/RFP	Δ*esaN* transformed with pFPV-*rfp*	This study
ΔFur box-P_*ethB*_::Km/RFP	ΔFur box-P_*ethB*_::Km transformed with pFPV-*rfp*	This study
Δ*esaN*ΔFur box box-P_*ethB*_::Km/RFP	Δ*esaN*ΔFur box-P_*ethB*_::Km transformed with pFPV-*rfp*	This study
*Plasmids*		
pRE112	Suicide plasmid, *pir* dependent, Cm^r^, *ori*T, *ori*V, *sacB*	[42]
pACYC-*escE*-*cyaA*	pACYC184 with *cyaA* fused to C terminus of *escE*; Cm^r^	[46]
pFPV-RFP	pFPV25.1, the g*fp* gene replaced by the *rfp* gene	[45]
pKD46	Red helper plasmid, Amp^r^	[44]
pKD4	template plasmid used to amplify FRT-flanked resistance gene, Amp^r^, Km^r^	[44]
pSU315	template plasmid with FLP recognition target site and 2HA tag sequence, Amp^r^, Km^r^	[47]
pSUB11	template plasmid with FLP recognition target site and 3FLAG tag sequence, Amp^r^, Km^r^	[47]
pKD46-*flp*	Red helper plasmid, Amp^r^	[48]

*aa* Amino acid, *Amp* ampicillin, *Cm* chloramphenicol, *Col* colistin, *Km* kanamycin, *LD*_*50*_ 50% lethal dose, *r* resistance,* RFP* red fluorescent protein, *s* sensitivity,* WT* wild type

### Cell lines, cultivation and bacterial infection

Epithelioma papillosum cyprini (EPC) cells were grown in M199 medium supplemented with 10% fetal bovine serum (FBS; Every Green) in an atmosphere containing 5% CO_2_ at 25 ℃. Murine J774A.1 macrophages were grown in Dulbecco’s minimal essential medium (DMEM) supplemented with 10% FBS (Gibco, Thermo Fisher Scientific) in a 5% CO_2_ atmosphere at 35 ℃. Infection of the EPC or J774A.1 cells with *E. piscicida* strains was performed as described by Xie et al. [5].

### Construction of mutant strains and plasmids

The primers used in this study (Table 2) were designed using the Primer Premier 5.0 software package (PREMIER Biosoft, Palo Alto, CA, USA). The Δ*ethA* strain was constructed using SacB-based allele exchange [42, 43]. Briefly, the upstream and downstream fragments flanking *ethA* were amplified using the *ethA*-for/*ethA*-int-rev and *ethA*-int-for/*ethA*-rev primer pairs, respectively. The PCR product was then cloned into the suicide vector pRE112 [42] and transformed into *Escherichia coli* S17-1 λ pir. Single-crossover mutants were obtained by conjugal transfer of the resulting plasmid into *E. tarda* PPD130/91. Double-crossover mutants were then selected on tryptic soy agar (TSA) plates containing 15% sucrose and 12.5 μg/mL colistin. The mutants were subsequently verified by PCR and sequencing.

**Table 2:** **Oligonucleotides used in this study**

Designation	Nucleotide sequence
ΔFur box-for	TAAAAACAGATTAGAAAGGATTAACTTTACGATGTTTTTTCGCCGTTCCATGCAGTGTAGGCTGGAGCTGCTTC
ΔFur box-rev	ACGACATCCCCCAGAGCGTTACCCATAGCAGGCAGAAAGGCGTTTTTTTATTCATATTATTCCCCCGGGCGACGGCAATGGGAATTAGCCATGGTCC
*ethA*-2HA for	AGCCATCCGGAGAGCAGCCAGAGCGGGGTTCACAGCAAACAG*TATCCGTATGATGTGCCGGACTATGCGTATCCGTATGATGTTCCTGAT*
*ethA*-2HA rev	ACTAAGGAGGATATTCATATGCGCGCACACCGCGTGCGCTCCGGACATTATTATTTTTTTGCA
*eseG*-3FLAG for	GAAAACCCGCGCGTTTCTCATTCAAACTCTCCGCCAAAACGGCGTCGAGTCT*GACTACAAAGACCATGACGGT*
*eseG*-3FLAG rev	CTGTGTATCGGGTGCATACCTGAATTGATGTGCGAGCGGCGCGCGGGCAT CATATGAATATCCTCCTTAGT
EthA_1-338 aa_::*cyaA*-for	CGGAATTCATCAGGGATGGCAGAGTGGCGT
EthA_1-338 aa_::*cyaA*-rev	AAAAGTACTTTAAGCGTAATCTGGAACATCGTATGGGTACCGATAGCTCTGCTCCCGG
*ethA*-for	GCTCTAGACGCTACAGCCGTGCCTACA
*ethA*-int-for	GCCGTCAGCGGCACAGGGTA
*ethA*-int-rev	TACCCTGTGCCGCTGACGGCAGAGGTTACCTCATATCATGATATGAGGTAACCTCT GCCGTCAGCGGCACAGGGTA
*ethA*-rev	GCTCTAGACGTTTCAAAGTGGGTATTCTGCAGGTGC

Underlining indicates the adapter sequence and italics indicate the HA or Flag tag sequence

*for* Forward,* rev* reverse

The λ Red recombination system [44] was used to delete the Fur box in front of *ethB* and tag the chromosomal copy of *ethA* with a 2HA epitope and chromosomal copy of *eseG* with a 3FLAG epitope. The homology arms used in the λ Red recombination system to construct bacterial strains range in length from 50 to 70 bp. For example, to delete the Fur box in front of *ethB*, the upstream homology arm corresponds to the 54-bp DNA fragment located immediately before the Fur box sequence, and the downstream homology arm to the 77-bp region located immediately after. The Fur box in front of *ethB* was replaced with the Km cassette to create the ΔFur box-P_*ethB*_::Km strain. This strategy involves replacing the chromosomal sequence of the Fur box in front of *ethB* with a Km selectable cassette generated by PCR using primers with homology extensions and adaptors (underlined in the primers shown in Table 2). In brief, the primer pair ΔFur box-for and ΔFur box-rev, using pKD4 as the template, were used to amplify the Km resistance gene. The PCR product was electroporated into the competent cells of the wild-type strain of *E. piscicida* PPD130/91 or the Δ*esaN* strain that had been introduced with the pKD46 plasmid. Induction with L-arabinose at 30 °C accomplishes λ Red-mediated recombination in these flanking homologies. The ΔFur box-P_*ethB*_::Km and Δ*esaN*ΔFur box-P_*ethB*_::Km strains were screened on TSA-Km plates and verified by PCR and sequencing.

The pFPV-RFP plasmid [45] was electroporated into the competent cells of the *E. piscicida* PPD130/91 wild-type (WT), Δ*esaN*, ΔFur box-P_*ethB*_, or Δ*esaN*ΔFur box*-P*_*ethB*_ strain to generate *E. piscicida* strains that express RFP.

The DNA fragment covering the N-terminal 338 amino acid residues of EthA, together with the ribosome binding site upstream of *ethA*, was digested with BamHI and BglII, before being inserted into pACYC-*escE*-*cyaA* [46] to replace *escE* and obtain pACYC-*ethA*_1-338 aa_::*cyaA*. Prior to electroporation into *E. piscicida* strains, the constructed plasmid was verified by DNA sequencing.

### Epitope tagging of the chromosomal *ethA *with a 2HA tag and *eseG* with a 3FLAG tag

To label the chromosomal copy of *ethA* with the 2HA epitope using the λ Red recombination system [47], the forward primer (*ethA*-2HA-for) covering the C-terminal sequence of *ethA* (excluding the stop codon), HA tag (the italics in the primer shown in Table 2) and the adaptor sequence (underlined sequence shown in Table 2) and the reverse primer (*ethA*-2HA-rev) covering another adaptor sequence (underlined sequence shown in Table 2) together with a chromosomal region downstream of *ethA* were used to amplify the Km cassette from pSU315 [47]. The PCR product was electroporated into *E. piscicida* PPD130/91 WT strain and Δ*esaN* strain, each of which was introduced with pKD46. The WT *ethA*-2HA::Km strain or Δ*esaN ethA*-2HA::Km strain was screened on TSA-Km plates and verified by immunoblotting with anti-HA antibodies. Similarly, ΔFur box-P_*ethB*_* ethA*-2HA::Km strain and Δ*esaN*ΔFur box-P_*ethB*_* ethA*-2HA::Km strain were constructed. It is worth noting that the pKD46-*flp* plasmid was electroporated into *E*. *piscicida* PPD130/91 ΔFur box-P_*ethB*_::Km strain or Δ*esaN*ΔFur box-P_*ethB*_::Km strain to eliminate the locus of FRT introduced by induction with L-arabinose at 30 °C and then the bacteria were cultured at 37 °C to eliminate the plasmid PKD46-*flp* [48], obtaining the ΔFur box-P_*ethB*_* ethA*-2HA strain and Δ*esaN*ΔFur box-P_*ethB*_* ethA*-2HA strain. In a similar way as described for HA tagging, the chromosomal copy of *eseG* was tagged with the 3FLAG epitope—with the exception that pSUB11 was the PCR template [47]—and the primers *eseG*-3FLAG-for and *eseG*-3FLAG-rev (Table 2) were used to amplify the Km cassette. The *E. piscicida* strains obtained were confirmed by immunoblotting with the anti-FLAG antibody.

### Immunoblotting

Overnight cultures of *E. piscicida* strains were diluted 1:200 in DMEM and grown without shaking in a 5% CO_2_ atmosphere at 25 ℃ for 24 h. Extracellular proteins (ECPs) and total bacterial pellets (TBPs) were prepared as previously described [49]. ECPs and TBPs were loaded onto a sodium dodeclyl sulphate-polyacrylamide gell electrophoresis (SDS-PAGE) gel, and electrophoresis using Tris–glycine running buffer was performed first at 70 V for 30 min, then at 120 V for 1 h, following which the separated proteins were transferred to polyvinylidene fluoride (PVDF) membrane (MilliporeSigma, Burlington, MA, USA) via wet transfer at 80 V for 1 h. The PVDF membrane was subsequently blocked for 1 h at room temperature with 5% (w/v) non-fat dry milk prepared in phosphate-buffered saline containing 0.1% Tween-20 (PBST). Following blocking, the membrane was incubated with rabbit antibodies against HA (1:2000; Cell Signaling Technology, Danvers, MA, USA), DnaK (1:1000, Cusabio, Houston, TX, USA), EseG (1:1000) [9] and EvpC (1:5000) [49] diluted in blocking buffer overnight at 4 °C.

J774A.1 cells were infected with *E. piscicida* strains at a multiplicity of infection (MOI) of 10. The cell culture supernatants were collected at 3 h post-infection (hpi), and the cells were lysed with 200 μL immunoprecipitation lysis buffer for 10 min on ice. The supernatants and cell lysates from each infection were precipitated with methanol and chloroform. The pellets were then resuspended in 1×  SDS loading buffer, the proteins separated by SDS-PAGE and then transferred to PVDF membranes. Membranes were probed with mouse anti-caspase-1 (p20) antibody (1:3000; AdipoGen, Füllinsdorf, Switzerland), rabbit anti-GSDMD antibody (1:3000; Abcam Ltd., Cambridge, UK), rabbit anti-caspase-3 monoclonal antibody (1:1000; Cell Signaling Technology), rabbit anti-cleaved caspase-3 (Asp175) monoclonal antibody (1:1000; Cell Signaling Technology), rabbit anti-MLKL (D6W1K) monoclonal antibody (1:3000; Cell Signaling Technology), rabbit anti-phosphorylated-MLKL (Ser345) monoclonal antibody (1:3000; Cell Signaling Technology), mouse anti-p65 (1:3000; Cell Signaling Technology), rabbit anti-phosphorylated-NF-κB p65 (Ser536) (93H1) monoclonal antibody (1:3000; Cell Signaling Technology), rabbit anti-TAK1(D94D7) monoclonal antibody (1:3000; Cell Signaling Technology), rabbit anti-phosphorylated TAK1 (Ser412) monoclonal antibody (1:3000; Cell Signaling Technology) and rabbit anti-actin polyclonal antibody (1:5000; ABclonal Technology, Woburn, MA, USA). The membranes were then incubated with horseradish peroxidase (HRP)-conjugated goat anti-rabbit IgG (1:3000; MilliporeSigma) or goat anti-mouse IgG (1:3000; MilliporeSigma) and then developed by HRP substrate (MilliporeSigma). Images were acquired using the ImageQuant LAS 4000 mini imaging system (GE HealthCare, Chicago, IL, USA).

The majority of the target proteins, along with the loading control, were electrophoresed, transferred and developed on the same PVDF membrane. For proteins with a similar molecular weight to the loading control (beta-actin), such as pro-caspase-1, sample aliquots were loaded on two parallel gels to ensure accurate normalization. Target protein levels were normalized to the loading control from the matched gel using ImageJ software.

### Translocation assay

The CyaA-based translocation assay was performed on EPC cells as previously described [9]. Briefly, EPC monolayers were infected at a MOI of 10 for 2 h with *E. piscicida* strains expressing the EthA_338aa_-CyaA fusion protein. Cyclic AMP (cAMP) levels were quantified by a cAMP enzyme-linked immunoassay (ELISA) according to the manufacturer’s instruction (Arbor Assays, Ann Arbor, MI, USA).

For the cell fractionation assay, EPC cells were seeded at 5 × 10^5^ cells per well in 24-well cell culture plates 1 day before infection. *Edwardsiella piscicida* cultures that reached 0.5 at OD_540_ were applied onto EPC monolayers at a MOI of 10 in M199 medium. The plates were centrifuged at 170 *g* for 5 min at room temperature and maintained for 30 min at 25 °C in an incubator with 5% CO_2_ atmosphere, the medium was replaced with M199 medium supplemented with 50 µg/mL gentamicin. At 2 h post-infection, EPC monolayers were washed once with PBS (phosphate-buffered saline) and lysed with 250 μL 0.2% Triton X-100 in PBS for 10 min on ice. Soluble (containing translocated effectors) and insoluble (containing bacteria and nucleus) fractions were separated by centrifugation (16 000 *g*, 10 min, 4 °C). The supernatant was mixed with 5× protein loading buffer and subjected to immunoblotting.

### In situ detection of fragmented DNA and p65 nuclear translocation in J774A.1 cells by immunofluorescence staining

Murin macrophage J77A.1 cells were seeded onto glass coverslips in 24-well plates 24 h prior to infection with *E. piscicida* strains expressing RFP as described previously [45]. Briefly, J774A.1 cells were infected for 3 h at an MOI of 10 with *E. piscicida* strains expressing RFP. Apoptotic levels in infected J774A.1 cells were assessed with the TUNEL (terminal deoxynucleotidyl transferase dUTP nick end labeling) assay using the DeadEnd™ Fluorometric TUNEL System (Promega; Madison, WI, USA) according to the manufacturer’s instructions. Images of positively infected cells were captured and scored for the number of TUNEL-positive cells using a Leica fluorescence microscope (DM4 B; Leica). For nuclear translocation of p65, J774A.1 cells on coverslips were stained with mouse anti-p65 (1:200, Cell Signaling Technology), Alexa 488-conjugated goat anti-mouse IgG (1:200, Invitrogen) and DAPI. The p65 nuclear translocation was recorded using a Leica fluorescence microscope (DM4 B; Leica, Wetzlar, Germany).

### Lactate dehydrogenase release measurement

J774A.1 cells were cultured in 24-well plates and infected with *E. piscicida* strains at a MOI of 10 for 3 h. Lactate dehydrogenase (LDH) assays were performed according to the manufacturer’s instructions (Roche, Basel, Switzerland) and detected at 490 nm using ELx800 microplate reader (BioTek Instruments, Winooski, VT, USA).

### Single-strain infection in zebrafish larvae

To infect the zebrafish larvae, 30 larvae per infection were immersed in 70-cm dishes containing 2.5 × 10^8^ colony-forming units (CFUs)/mL of the *E. piscicida* strain on day 4 post-fertilization (dpf) for 6 h. Then, 20 mL of water was added to maintain the infection at 28 °C. Mortality was recorded over a period of 3 days. Additionally, ten zebrafish larvae per infection were pooled to create a sample for the bacterial load assay. At the indicated time post-infection, the samples were homogenized and serially diluted before being plated onto TSB agar plates to count the CFUs.

### Statistical analysis

All data are presented as the mean ± standard error of the mean (SEM) or as the mean ± standard deviation (SD). Significance was analyzed using a two-tailed Student’s t-test or a one-way analysis of variance (ANOVA; Duncan’s multiple range test). Statistical tests were applied to data from at least three independent experiments, or from one representative experiment. *P* values < 0.05 were considered to be statistically significant.

## Results

### The hemolysin EthA is secreted and translocated mainly in a T3SS-dependent manner

**Figure 1:**
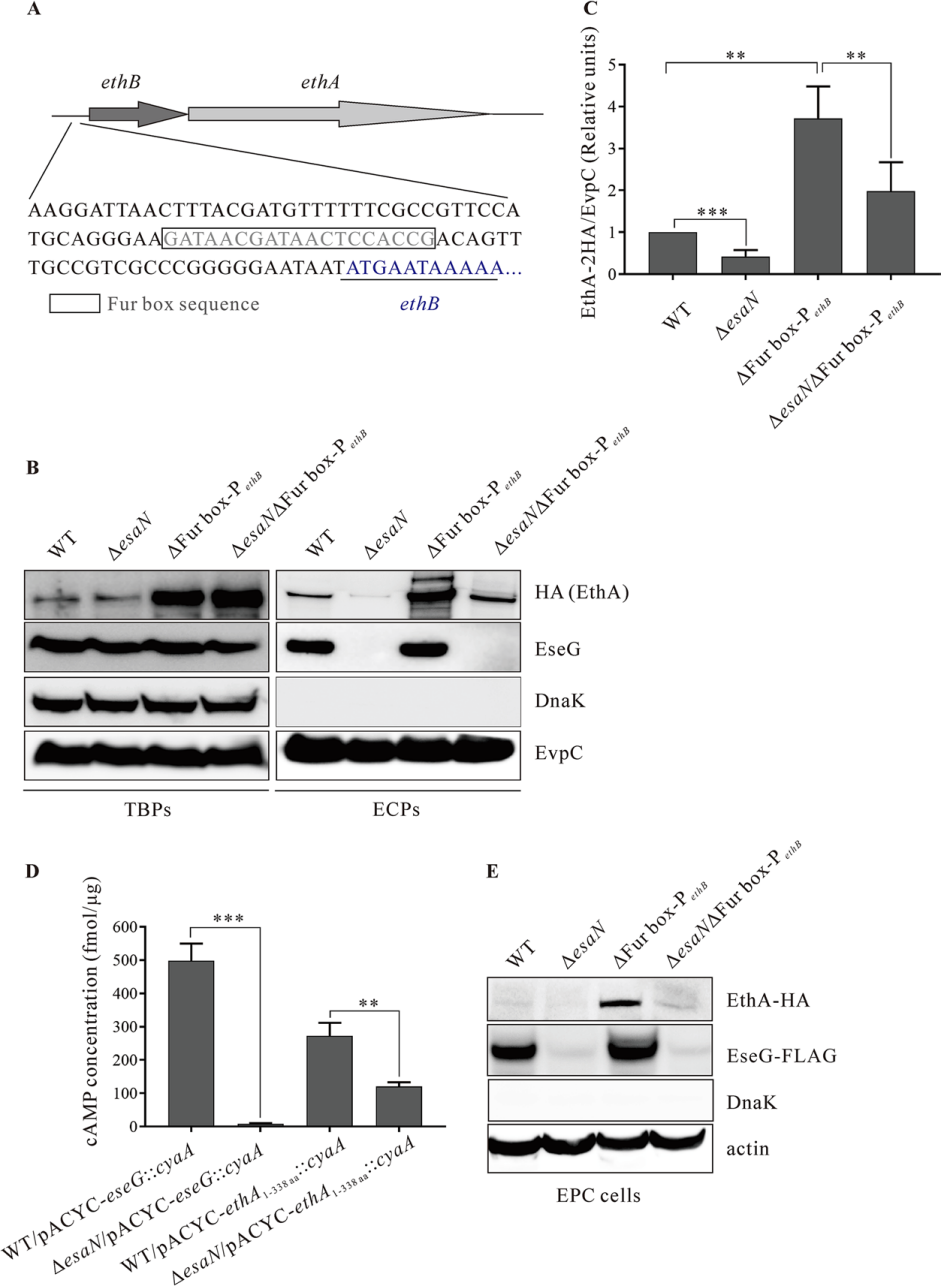
**The hemolysin EthA is secreted and translocated in a T3SS-dependent manner.** **A** Nucleotide sequence upstream of the *ethB* gene in *Edwardsiella piscicida* PPD130/91. The DNA fragment of the Fur binding box (Fur box) is framed. The *ethB* gene is underlined with a solid line. **B** EthA is primarily secreted into the culture supernatant via the type III secretion system (T3SS). Similar amounts of protein in TBPs or ECPs from the WT *ethA*-2HA, Δ*esaN ethA*-2HA, ΔFur box-P_*ethB*_* ethA*-2HA and Δ*esaN*ΔFur box-P_*ethB*_* ethA*-2HA strains were probed with anti-HA (EthA), anti-EseG (T3SS effector), anti-DnaK (cytosolic marker) and anti-EvpC (T6SS cargo protein) antibodies. **c** Quantitative analysis of secreted EthA from different *E. piscicida* strains shown in Figure 1B (right panel). EthA levels were quantified by densitometry and normalized to those of EvpC. The graph shows the ratio of EthA (mean ± standard error of the mean [SEM]) from three independent experiments. Asterisks indicate significant difference at **P < 0.01 and ***P < 0.001, two-tailed Student’s *t*-test. **D** EthA is partially translocated into the host cell via the T3SS. The EPC monolayers were infected with *E.piscicida* strains transformed with pACYC-*eseG*::*cyaA* or pACYC-*ethA*_1-338 aa_::*cyaA* for 2 h, and the EPC monolayers were lysed to measure the intracellular cAMP levels by using a cAMP enzyme-linked immunoassay. The graph shows the mean ± standard deviation (SD) of a representative experiment. Asterisks indicate significant difference at **P < 0.01 and ***P < 0.001, one-way analysis of variance (ANOVA; Duncan’s multiple range tests). **E** EthA translocation examined by cell fractionation and immunoblotting. EPC monolayers were infected for 2 h before fractionation with Triton-X 100. The Triton-X 100 soluble fraction of the infected EPC cells was analyzed by immunoblotting using anti-HA (EthA), anti-FLAG (EseG), anti-DnaK and anti-actin antibodies. The Triton-X 100 soluble fraction from EPC cells contains translocated EthA.* cAMP* Cyclic adenosine monophosphate, * ECPs* extracellular proteins,* HA* hemagglutinin, *TBPs* total bacterial pellets,* WT* wild type, EPC cells Epithelioma papillosum cyprini (EPC) cells.

Several T3SS effector candidates, including EthA, have been identified by comparing the secretomes of the Δ*esaB* and Δ*esaB*Δ*esaN* strains [18]. A Fur-binding DNA motif GATAACGATAACTCCACC (Fur box) (Figure 1A), where Fur-Fe^2+^ binds in the 5′- upstream region of the *ethB* gene, was deleted using the one-step chromosomal gene inactivation [44], obtaining the ΔFur box-P_*ethB*_ strain and the Δ*esaN*ΔFur box-P_*ethB*_ strains. To verify the T3SS-dependent secretion of EthA, the *ethA* gene in WT, Δ*esaN*, ΔFur box-P_*ethB*_ and Δ*esaN*ΔFur box-P_*ethB*_ strains was chromosomally tagged with the 2HA epitope using the λ Red recombination system [47]. Significantly higher steady-state protein levels of EthA-2HA were detected by immunoblotting in the TBPs of the ΔFur box-P_*ethB*_ strain and Δ*esaN*ΔFur box-P_*ethB*_ strain, in stark contrast to its counterpart in the WT or Δ*esaN* strain, where they were barely detectable (Figure 1B, left panel). Inactivation of the T3SS (EsaN depletion) abolishes the secretion of EseG and markedly decreases the secretion of EthA (Figure 1B, right panel, C). As EseG and EvpC are the T3SS and T6SS substrates [9, 49], they were used as the loading controls. The cytosolic chaperone DnaK was not detected in the ECPs, indicating that the EthA present in ECPs is not leakage from bacterial pellets. These results suggest that EthA is primarily secreted through the T3SS.

The authors of an earlier study reported that EthA enters host cells via dynamin-dependent endocytosis through binding to OMVs [29]. As EthA is secreted mainly via the T3SS, it is possible that it also enters host cells via this same delivery method. To investigate this possiblility, we introduced the reporter plasmid pACYC-*ethA*_1-338 aa_::*cyaA* into both the WT and the Δ*esaN* strains. When the N-terminal fusion of the effector protein to CyaA is introduced into a eukaryotic cell, the calmodulin-activated CyaA protein converts adenosine triphosphate (ATP) into cAMP, and the increase in cAMP is then measured to demonstrate the translocation of the effector protein [50]. In the present study, EPC monolayers were infected with *E. piscicida* strains, and the level of cAMP inside the EPC monolayers was measured at 2 hpi as an indicator of EthA translocation. The cAMP levels were 272.8 ± 38.87 fmol/μg in WT/pACYC-*ethA*_1-338 aa_::*cyaA-*infected cells; in contrast, significantly lower levels of cAMP (120.8 ± 12.37 fmol/μg) were observed in the Δ*esaN* /pACYC-*ethA*_1-338 aa_::*cyaA-*infected cells. The cAMP level in WT/pACYC-*eseG*::*cyaA*-infected cells was examined as a positive control (497.6 ± 52.17 fmol/μg protein), and found to be significantly higher than the cAMP level in the negative control Δ*esaN*/pACYC-*eseG*::*cyaA*-infected cells (8.369 ± 1.75 fmol/μg protein) (Figure 1D). These results suggest that EthA is primarily translocated into host cells via the T3SS.

To corroborate this result, EPC monolayers were infected for 2 h with *E. piscicida* strains that chromosomally express both EseG-3FLAG and EthA-2HA. The translocated proteins were then extracted from the post-nuclear supernatant using Triton X-100 and analyzed by immunoblotting. Translocated EthA was easily be detected in cells infected with the ΔFur box-P_*ethB*_ strain background. A weak EthA band was visible in cells infected with the WT strain background or Δ*esaN*ΔFur box-P_*ethB*_ strain background. As expected, EthA translocation was barely detectable upon infection with the Δ*esaN* strain background (Figure 1E). These results verify that EthA is primarily translocated via the T3SS injectisome.

Taken together, these results suggest that hemolysin EthA is a T3SS substrate whose secretion and translocation depend primarily on an active T3SS.

### The T3SS-translocated EthA induces PANoptosis in murine macrophages

**Figure 2:**
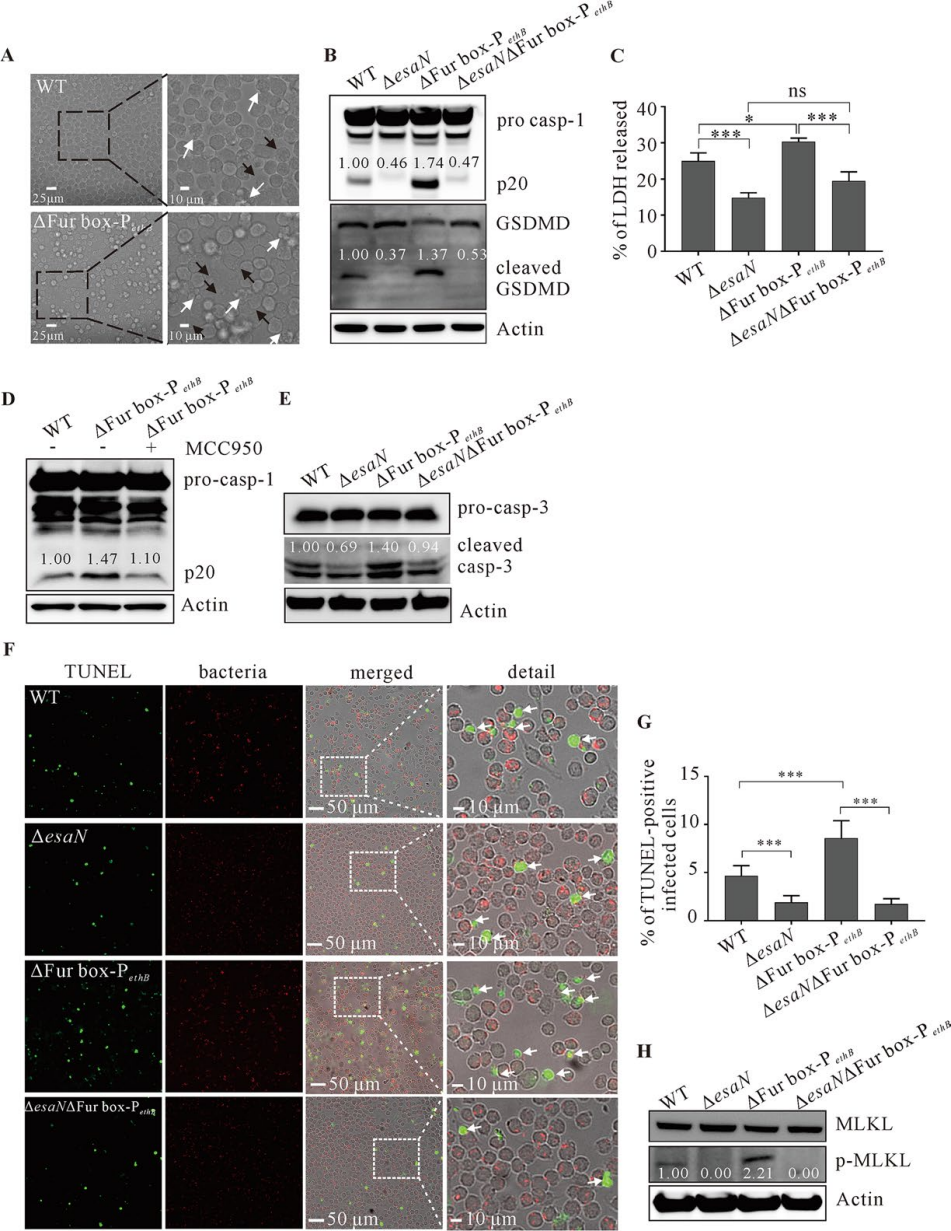
**The hemolysin EthA stimulates PANoptosis (a combination of pyroptosis, apoptosis and necroptosis) in J774A.1 cells.** **A** Morphology of J774A.1 cells infected with the WT strain and the ΔFur box-P_*ethB*_ strain. Black arrows indicate cell swelling and white arrows indicate cell blebbing. Scale bars: 10 or 25 μm. **B** The immunoblotting of cleaved caspase-1 (*p20*) and cleaved GSDMD in J774A.1 cells infected with *Edwardsiella piscicida* strains. **C** LDH released by J774A.1 cells infected with *E. piscicida* strains. **D** EthA induces pyroptosis partially via the NLRP3 inflammasome pathway. J774A.1 cells were treated with 700.0 μM MCC950 (NLRP3 inhibitor) for 2 h prior to infection with WT and ΔFur box-P_*ethB*_ strains. **E** Immunoblotting of pro-caspase-3 and cleaved caspase-3 in J774A.1 cells infected with *E. piscicida* strains. **F** TUNEL-stained J774A.1 cells infected with *E. piscicida* strains. White arrows indicate fragmented nuclei. Scale bars: 50 or 10 µm. **G** Percentage of TUNEL-positive cells in J774A.1 cells positively infected with *E. piscicida* WT/RFP, Δ*esaN*/RFP, ΔFur box-P_*ethB*_/RFP, and Δ*esaN*ΔFur box-P_*ethB*_/RFP strains. **H** Immunoblotting of the steady-state protein levels of MLKL and phophorylated (p)-MLKL in J774A.1 cells infected with *E. piscicida* strains. The levels of target protein were quantified using densitometry and normalized against the levels of actin. The numbers on the bands indicate the relative intensity of p20, cleaved-GSDMD, cleaved caspase-3 or p-MLKL, obtained by normalizing WT to a relative intensity of 1. Data are representative of those from three independent experiments. The significance was analyzed using the two-tailed Student’s *t*-test (**C**) or one-way analysis of variance (ANOVA; Duncan’s multiple range tests) (**G**). Asterisks indicate signifcant difference at *P < 0.05 and ***P < 0.001; *ns* not significant.* GSDMD* Gasdermin D,* LDH* lactate dehydrogenase,* MLKL* mixed lineage kinase domain-like protein,* RFP* red fluorescent reporter protein,* TUNEL* terminal deoxynucleotidyl transferase dUTP nick end labeling,* WT* wild type.

EthA, which enters host cells via its association with OMVs, induces cell membrane rupture in epithelial cells, such as Caco-2 or HeLa cells [29]. To investigate the phenotype of EthA in murine macrophages after translocation via the T3SS, we infected J774A.1 cells with the *E. piscicida* WT and ΔFur box-P_*ethB*_ strains at an MOI of 10 for 3 h. Higher ratios of cell swelling and blebbing occurred in J774A.1 cells infected with the ΔFur box-P_*ethB*_ strain than in those infected with the WT strain (Figure 2A). Cell swelling, activation of caspase-1 and membrane rupture are the key features of pyroptosis [51]. Infection of the J774A.1 murine macrophage cell line with the ΔFur box-P_*ethB*_ strain resulted in significantly higher steady-state protein levels of p20 (cleaved caspase-1) than did infection with the WT strain; p20 was barely detectable in J774A.1 cells infected with either the Δ*esaN* strain or the Δ*esaN*ΔFur box-P_*ethB*_ strain (Figure 2B). Cleaved caspase-1 cleaves gasdermin D (GSDMD) [34]. As expected, increased levels of cleaved GSDMD were observed in J774A.1 cells infected with the ΔFur box-P_*ethB*_ strain compared those infected with the WT strain. Cleaved GSDMD was barely detectable in cells infected with the Δ*esaN* strain or the Δ*esaN*ΔFur box-P_*ethB*_ strain (Figure 2B). The N-terminal fragment of cleaved GSDMD forms a pore in the host cell membrane, allowing LDH to leak out. Therefore, the release of LDH was examined. As expected, significantly higher levels of LDH were detected in the culture supernatant of J774A.1 cells infected with the ΔFur box-P_*ethB*_ strain than in that infected with the WT strain. Lower levels were detected with the Δ*esaN* strain and the Δ*esaN*ΔFur box-P_*ethB*_ strain (Figure 2C). This result indicates that cell rupture was induced by T3SS-translocated EthA. Next, J774A.1 monolayers were pre-treated with 700 μM of the MCC950 drug before being infected with *E. piscicida* strains. The MCC950 inhibitor disrupts the oligomerization and activation of the NLRP3 inflammasome [52]. This pre-treatment inhibited caspase-1 cleavage in macrophages infected with the ΔFur box-P_*ethB*_ strain by a small but significant amount (Figure 2D). Taken together, these results demonstrate that the T3SS-translocated EthA induces pyroptosis, a process that is partially mediated by the NLRP3 inflammasome pathway.

Cell blebbing is one of the defining features of apoptosis [53]. Cleaved caspase-3 is an executioner caspase of apoptosis [36]. We found the infection with the ΔFur box-P_*ethB*_ strain dramatically and significantly elevated the steady-state protein levels of cleaved caspase-3 in J774A.1 monolayers as compared to the WT, Δ*esaN* or Δ*esaN*ΔFur box-P_*ethB*_ strain (Figure 2E). DNA fragmentation is another hallmark of apoptosis [54]. We performed TUNEL assays on J774A.1 cells infected with *E. piscicida* strains expressing RFP and found that 8.59 ± 0.54% of the J774A.1 cells were TUNEL-positive following infection with the ΔFur box-P_*ethB*_/RFP strain. By contrast, the infection rate was significantly lower with the WT/RFP strain (4.67 ± 0.3%), Δ*esaN*/RFP strain (1.9 ± 0.2%) and Δ*esaN*ΔFur box-P_*ethB*_/RFP strain (1.73 ± 0.17%) (Figure 2F, G). These results demonstrate that EthA, which is translocated via the T3SS, induces apoptosis in murine macrophages.

Necroptosis is characterized by cell rupture and organelle swelling, and is a form of programmed cell death that does not involve caspases [37]. The MLKL protein acts as the executor of necroptosis by forming pores in the cell membrane [55]. When similar levels of total MLKL protein were detected in J774A.1 cells infected by different strains, the level of phosphorylated MLKL had increased significantly in J774A.1 cells infected with the ΔFur box-P_*ethB*_ strain as compared to the WT strain; in addition, phosphorylated MLKL was barely detectable in cells infected with either the Δ*esaN* strain or the Δ*esaN*ΔFur box-P_*ethB*_ strain (Figure 2H). These results demonstrate that T3SS-translocated EthA stimulates necroptosis in murine macrophages.

Taken together, these results show that significantly higher levels of cleaved caspase-1, cleaved caspase-3 and phosphorylated MLKL are stimulated by the ΔFur box-P_*ethB*_ strain as compared to the Δ*esaN*ΔFur box-P_*ethB*_ strain, indicating that the translocation of EthA via the T3SS induces pyroptosis, apoptosis and necroptosis, thereby reflecting the stimulation of PANoptosis.

### The T3SS-translocated EthA inhibits the NF-κB signaling pathway

**Figure 3:**
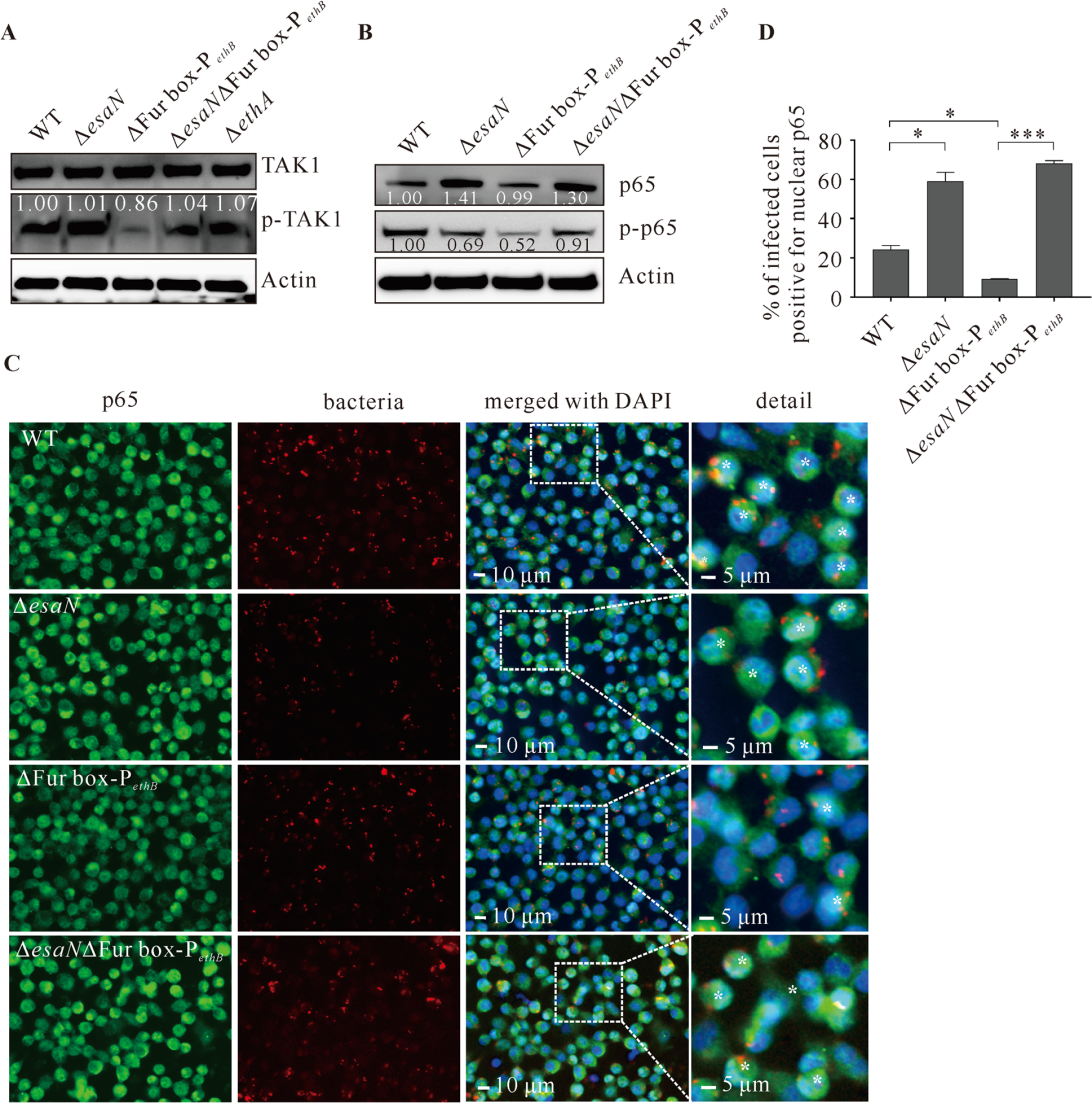
**The hemolysin EthA suppresses the TAK1/NF-κB signaling pathway.** **A** Immunoblotting of steady-state protein levels of TAK1 and phosphorylated (p)-TAK1 in J774A.1 cells infected with *Edwardsiella piscicida* strains. **B** Immunoblotting was used to analyze the steady-state protein levels of total p65 and p-p65 in J774A.1 cells infected with different *E. piscicida* strains. The numbers beneath the bands in 3A and 3B indicate the relative intensity of p-TAK1, p65 or p-p65, each of which was calculated by normalizing WT to a relative intensity of 1. **C** Immunofluorescence staining with a mouse anti-p65 antibody and Alexa 488-labeled goat anti-mouse IgG were used to examine the nuclear accumulation of transcription factor p65 (green) in J774A.1 cells infected with the *E. piscicida* WT/RFP, Δ*esaN*/RFP, ΔFur box-P_*ethB*_/RFP, and Δ*esaN*ΔFur box-P_*ethB*_/RFP strains at a multiplicity of infection (MOI) of 10 for 3 h. The *E. piscicida* strains are indicated by RFP. Asterisks indicate the location of p65 accumulation in the nucleus of infected cells. Scale bars: 10 or 5 µm. **D** The percentage of p65-positive cells in the nucleus of J774A.1 cells infected with different *E. piscicida* strains. The levels of target protein were quantified using densitometry and normalized against the levels of actin. Data are representative of data from three independent experiments. Significance was analyzed using one-way analysis of variance (ANOVA; Duncan’s multiple range tests) **D** Asterisks indicate signifcant difference at *P < 0.05 and ***P < 0.001.* RFP* Red fluorescent reporter protein,* TAK1/NF-κB* transforming growth factor β-activated kinase 1/nuclear factor κB,* WT* wild type.

Activation of the TAK1-dependent signaling pathway requires that TAK1 undergo phosphorylation [56]. Inhibiting or deleting TAK1 leads to the assembly of the PANoptosome [57]. As EthA promotes PANoptosis in murine macrophages, might EthA also play a role in TAK1 phosphorylation? In an attempt to answer this question, J774A.1 monolayers were infected with *E. piscicida* strains at an MOI of 10 for 3 h, following which the cell lysates and culture supernatants were precipitated for immunoblotting against TAK1 and phosphorylated TAK1 (Ser412). Similar levels of total TAK1 protein were detected in each infection; however, infection with the ΔFur box-P_*ethB*_ strain resulted in a significant and dramatic reduction in steady-state p-TAK1 protein levels compared to infection with the WT, Δ*esaN*, Δ*esaN*ΔFur box-P_*ethB*_ or Δ*ethA* strain (Figure 3A), suggesting that T3SS-translocated EthA inhibits TAK1 phosphorylation. Next, the steady-state protein levels of total p65 and phosphorylated p65 (p-p65) (Ser536) were examined and quantified in J774A.1 macrophages infected with *E. piscicida* strains. Significantly higher levels of total p65 were detected in J774A.1 cells infected with the Δ*esaN* or Δ*esaN*ΔFur box-P_*ethB*_ strain, whereas similarly lower levels were detected in cells infected with the WT or ΔFur box-P_*ethB*_ strain (Figure 3B). However, the lowest levels of p-p65 protein were detected in J774A.1 cells infected with the ΔFur box-P_*ethB*_ strain, with the Δ*esaN* strain having the second to lowest level (Figure 3B). Phosphorylated RelA/p65 usually moves into the nucleus, where it activates the NF-κB signaling pathway [58]. Using an immunofluorescence staining assay, we found that 9.31 ± 0.08% of cells infected with the ΔFur box-P_*ethB*_ strain underwent p65 nuclear translocation; this is significantly lower than the 24.27% ± 1.19% observed in cells infected with the WT strain (Figure 3C, D). Interestingly, significantly higher ratios were detected in samples infected with either the Δ*esaN* strain (59.08% ± 2.60%) or the Δ*esaN*ΔFur box-P_*ethB*_ strain (68.18% ± 0.81%) (Figure 3D). These results imply that the T3SS-translocated EthA protein prevents the accumulation of p65 in the nucleus of murine macrophages.

Overall, the T3SS-translocated EthA protein suppresses the phosphorylation of TAK1 and inhibits the NF-κB signaling pathway.

### EthA contributes to the fitness of *E. piscicida* in in vivo

**Figure 4:**
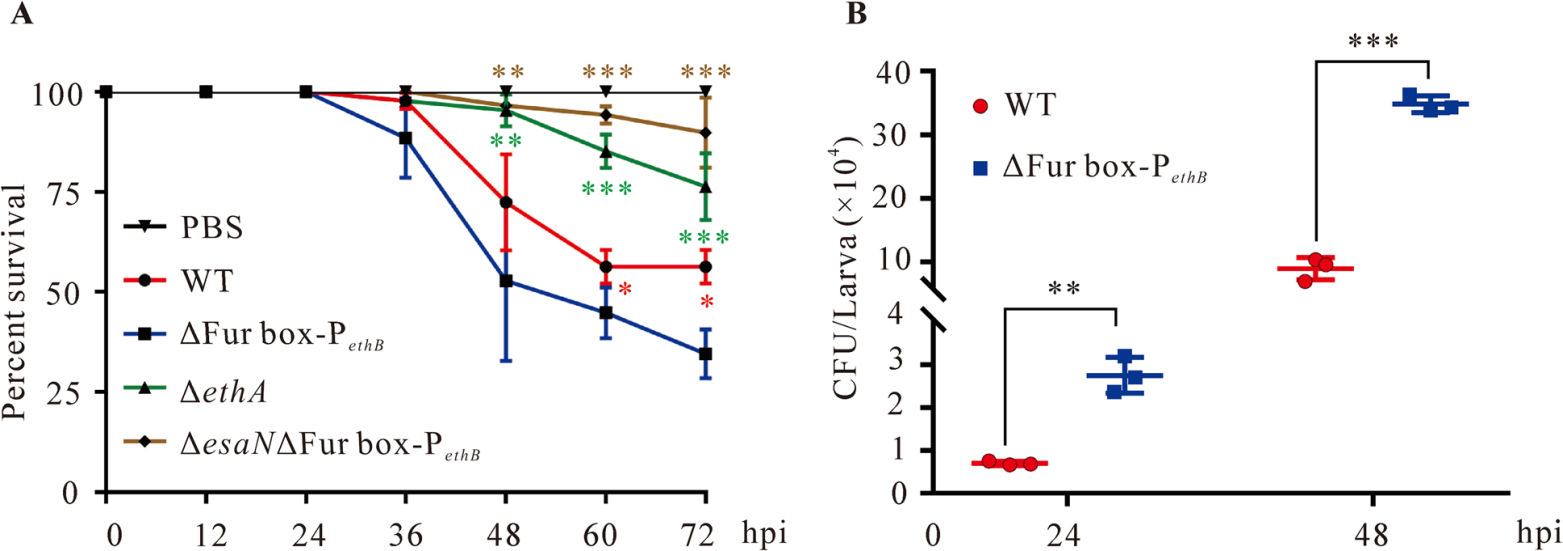
**The hemolysin EthA contributes to the virulence of *Edwardsiella piscicida* in fish. A** Four-day-old zebrafish larvae were infected by immersion with the *E. piscicida* WT, Δ*ethA*, ΔFur box-P_*ethB*_ or Δ*esaN*ΔFur box-P_*ethB*_ strain, respectively. The survival of the zebrafish larvae was monitored for 72 h, and the graph is based on three repeats of the experiment. Asterisks (**A**) indicate a significant difference in survival at *P < 0.05, **P < 0.01, ***P < 0.001, one-way analysis of variance (ANOVA; Duncan’s multiple range tests). Color coding of asterisks: red, WT strain vs ΔFur box-P_*ethB*_ strain; green, Δ*ethA* strain vs ΔFur box-P_*ethB*_ strain; brown, Δ*esaN*ΔFur box-P_*ethB*_ strain vs ΔFur box-P_*ethB*_ strain. **B** Ten zebrafish larvae infected with the WT or ΔFur box-P_*ethB*_ strain were pooled as one sample at the indicated time point post-infection. The number of bacteria load was determined by CFU counts. Asterisks (**B**) indicate a significant difference at **P < 0.01, ***P < 0.001, two-tailed Student’s t-test.* CFU* Colony-forming units,* PBS* phosphate buffered saline,* WT* wild type.

To assess the contribution of EthA in *E. piscicida* virulence, we performed survival assays in zebrafish larvae. Briefly, 4-day-old zebrafish larvae were infected by immersion in a solution containing 2.5 × 10^8^ CFU/mL of *E. piscicida* strain for 6 h and maintained for 3 days. The survival rate of those infected with the Δ*ethA* strain was significantly higher (76.27%) than that infected with the WT strain (56.29%). In contrast, the survival rate was significantly lower for larvae infected with the ΔFur box-P_*ethB*_ strain (34.51%) (Figure 4A). Consistently, zebrafish larvae infected with the ΔFur box-P_*ethB*_ strain exhibited a higher bacterial load at 24 and 48 hpi than those infected with the WT strain (Figure 4B). These results lead to the conclusion that EthA improves the fitness of *E. piscicida *in vivo.

## Discussion

The hemolysin EthA in *E. piscicida* binds to OMVs to facilitate its own translocation. The lipopolysaccharide (LPS) released by EthA from the OMVs then induces pyroptosis in HeLa cells [29]. In addition to the OMV pathway through which EthA can be translocated, our study demonstrates that EthA can also be secreted and translocated in a T3SS-dependent manner. The T3SS-translocated EthA protein suppresses the phosphorylation of TAK1, thereby inducing PANoptosis in murine macrophages by promoting pyroptosis, apoptosis and necroptosis, while inhibiting the NF-κB signaling pathway. This research sheds light on the novel strategy employed by *E. piscicida* to promote infection by using the cell-associated hemolysin EthA.

The significantly lower secretion of EthA from the Δ*esaN*ΔFur box-P_*ethB*_ strain compared to the ΔFur box-P_*ethB*_ strain suggests that EthA is secreted mainly via the T3SS. This result is consistent with the findings in *V. parahaemolyticus*, where the TDH hemolysin is cleaved in the periplasm by leader peptidase, and the truncated mature TDH is then imported and secreted in a T3SS-dependent manner [30]. However, differently, we found that deleting the N-terminal 1–29 amino acids (the Sec signal) from EthA results in instability and failure to be secreted (data not shown). As the molecular weight of secreted EthA is similar to that of its intracellular counterpart, it would appear that full-length EthA is secreted via the T3SS in *E. piscicida*. EthA is the second hemolysin that has been verified as being secreted via the T3SS. Further research is needed to establish whether TPS hemolysins usually undergo translocation via the T3SS.

Studies investigating the role of TPS hemolysin in cell death have used bacterial strains that overexpress hemolysin. For example, ExlA^+^ *P. aeruginosa* (*P. aeruginosa* that overexpresses ExlA) activates the NLRP3 inflammasome, inducing inflammatory pyroptotic death in macrophages [31]. Similarly, ShlA^+^ *S. marcescens* induces caspase-dependent death of macrophages, which can be prevented by pretreatment with the pan-caspase inhibitor Z-VAD-FMK [31]. In this study, the Fur box motif in front of the *ethB-ethA* operon was deleted to create the EthA^+^ strain of *E. piscicida* (ΔFur box-P_*ethB*_). This strain produced extremely high levels of cleaved caspase-1, cleaved caspase-3 and p-MLKL in J774A.1 macrophages. However, the Δ*ethA* strain exhibits characteristics similar to those of the WT strain with regard to the induction of pyroptosis, apoptosis and necroptosis in J774A.1 macrophages (Additional file 1A–D)*.* These findings are consistent with previous reports on the *E. piscicida* 0909I strain (EIB202, the parent strain) and the 0909IΔ*ethA* strain. The 0909I strain overexpresses EthA due to transposon insertion disrupting the operator of the *ethB-ethA* operon, inducing a form of cell death in the liver of zebrafish that depends on caspase-5-like activity and resembles pyroptosis [59]. That strain activates a non-canonical inflammasome via the caspase-4/11 pathway in human intestinal epithelial cell lines [29]. Furthermore, it induces significant membrane rupture in neutrophils through the action of caspase B (Caspy2) and GSDME [60]. However, consistent with the results of this study, no difference in caspase-4 processing was observed between the WT of *E. piscicida* EIB202 strain and the 0909IΔ*ethA* mutant [29].

We observed significant attenuation in zebrafish larvae infected with the Δ*ethA* strain, compared to those infected with the WT PPD130/91 strain. *Edwardsiella piscicida* is an enteric pathogen, and it has beens hypothesized that EthA expression is stimulated by Fe^2+^ scarcity, indole or other environmental cues in the gastrointestinal tract of fish, which differ from the conditions in a cell culture. In the latter case, EthA expression may not be strongly induced. Cell death may only be triggered when the level of EthA protein reaches a certain threshold, which may explain the weak difference in cell death stimulation by the *E. piscicida* WT and Δ*ethA* strains. Hemolysins play an important role in extra-intestinal dissemination [61, 62], and this role is supported by the observation that overexpression of EthR, the negative regulator of EthA, prevents dissemination of *E. piscicida* [63]. Therefore, deleting *ethA* impairs the ability of the strain to lyse host cells and cause tissue damage, thereby preventing dissemination. This explains the significant difference in survival rates observed when comparing infection with the Δ*ethA* strain and the WT strain of *E. piscicida* PPD130/91. In contrast, in a previous study, the survival rate of fish infected with the 0909IΔ*ethA* strain was slightly higher than that of the WT EIB202 strain, in both larvae and adults [59]. One explanation may be that *E. piscicida* EIB202 has two TPS hemolysin systems (ETAE-0820/0821 and ETAE-0910/0911), whereas PPD130/91 only has one. The functional redundancy of hemolysins in *E. piscicida* EIB202 may explain why EthA contributes differently to the virulence of the PPD130/91 and EIB202 strains of *E. piscicida*.

EthA inhibits the phosphorylation of both TAK1 and p65. TAK1 acts as a central hub that regulates both PANoptosis and the NF-κB signaling pathway [64]. Activation of the IκB kinase (IKK) complex by phosphorylated TAK1 results in the phosphorylation and subsequent degradation of IκB. This, in turn, results in the nuclear translocation of p65 and the activation of the NF-κB signaling pathway [65]. Interestingly, steady-state protein levels of total p65 increased significantly in J774A.1 cells infected with the Δ*esaN* or Δ*esaN*ΔFur box-P_*ethB*_ strain, compared to those infected with the WT or ΔFur box-P_*ethB*_ strain. Meanwhile, comparable transcriptional levels of p65 were detected in J774A.1 macrophages infected with any of the aforementioned four strains (data not shown). The elevated levels of total p65, stimulated by the Δ*esaN* background strain, may be due to the promotion of p65 degradation by *E. piscicida* T3SS effector EseH via ubiquitination [14]. Conversely, the T3SS-translocated EthA modulates the NF-κB signaling pathway by inhibiting p65 phosphorylation rather than influencing its degradation.

A major limitation to this study is that the murine macrophage cell line J774A.1 was used to investigate PANoptosis induced by the hemolysin EthA in *E. piscicida* because the necessary fish antibodies for the PANoptosis pathway are currently unavailable and a fish macrophage cell line is not yet available. We plan to validate our findings using fish macrophages once the necessary antibodies are available.

**Figure 5:**
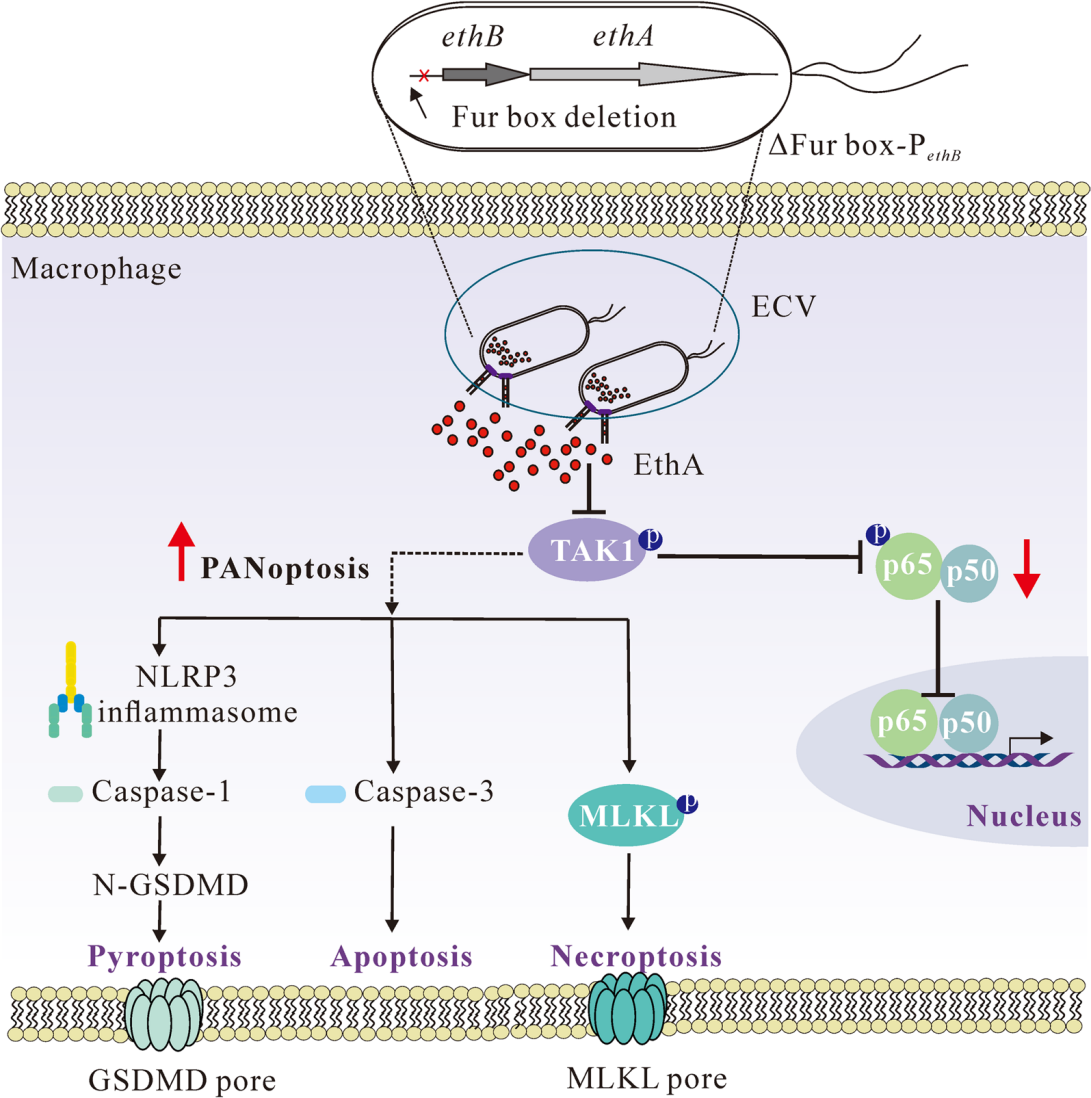
**Schematic illustration of the hemolysin EthA translocation pathway and EthA-triggered PANoptosis (a combination of pyroptosis, apoptosis and necroptosis) in murine macrophages.** The *E. piscicida* ΔFur box-P_*ethB*_ strain expresses high levels of the hemolysin EthA and utilizes the T3SS system to deliver EthA into the host cell cytosol via ECVs. The EthA protein, which is translocated via the T3SS, inhibits the phosphorylation of TAK1. This induces pyroptosis by partially activating the NLRP3 inflammasome, and provokes apoptosis and necroptosis by activating caspase-3 and phosphorylating MLKL, respectively. Together, these processes indicate the occurrence of PANoptosis in the macrophages. Meanwhile, translocating EthA inhibits the phosphorylation of p65 and blocks its nuclear translocation and the NF-κB signaling pathway in murine macrophages.* ECVs* *Edwardsiella*-containing vacuoles,* MLKL* mixed lineage kinase domain-like protein, *NF-κB* nuclear factor κB, *TAK1* transforming growth factor β-activated kinase 1,* T3SS* type III secretion system.

In summary, the hemolysin EthA in *E. piscicida* can be delivered into host cells via the T3SS. Once translocated, EthA inhibits the phosphorylation of TAK1, which is a key negative regulator of PANoptosis. Consequently, EthA stimulates PANoptosis in macrophages and inhibits the phosphorylation of the p65 protein (Figure 5). The novel T3SS effector EthA improves the fitness of *E. piscicida* by suppressing the inflammatory signaling pathway, thereby facilitating replication in vivo. Our findings enhance our understanding of the pathogenic mechanisms of hemolysins produced by different bacterial pathogens.

## Supplementary Information

**Additional file 1 Depletion of EthA did not affect the levels of PANoptosis stimulated by** ***E. piscicida*** **PPD130/91in J774A.1 macrophages.** (A) Immunoblotting of cleaved caspase-1 (p20), cleaved caspase-3 and p-MLKL in J774A.1 cells infected with the E. piscicida WT, ΔeseJ and ΔethA strains. The cell lysates and culture supernatants were precipitated for probing. Actin was used as a loading control to indicate similar amounts of protein loading per lane. (B, C & D) Quantitative analysis of p20 (B), cleaved caspase-3 (C), and p-MLKL (D) in J774A.1 cells infected with the E. piscicidastrains shown in panel 1A. The levels of p20, cleaved caspase-3 and p-MLKL were quantified using densitometry and normalized to actin levels. The graph shows the relative ratios of p20, cleaved caspase-3, or p-MLKL obtained from three independent experiments. ***, *P*< 0.001; **, *P*< 0.01; *, *P*< 0.05; ns, not significant.

## Data Availability

No datasets were generated or analyzed during the current study.
