# Supplementary material for: Hemolysin EthA is an Edwardsiella T3SS effector that induces PANoptosis in macrophages
Source: Vet Res. 2026 Jul 11;57:130. doi: 10.1186/s13567-026-01814-x (PMC13355344; doi:10.1186/s13567-026-01814-x)
Supplement: Supplementary file 1 — Additional file 1 Depletion of EthA did not affect the levels of PANoptosis stimulated by E. piscicida PPD130/91in J774A.1 macrophages. (A) Immunoblotting of cleaved caspase-1 (p20), cleaved caspase-3 and p-MLKL in J774A.1 cells infected with the E. piscicida WT, ΔeseJ and ΔethA strains. The cell lysates and culture supernatants were precipitated for probing. Actin was used as a loading control to indicate similar amounts of protein loading per lane. (B, C & D) Quantitative analysis of p20 (B), cleaved caspase-3 (C), and p-MLKL (D) in J774A.1 cells infected with the E. piscicidastrains shown in panel 1A. The levels of p20, cleaved caspase-3 and p-MLKL were quantified using densitometry and normalized to actin levels. The graph shows the relative ratios of p20, cleaved caspase-3, or p-MLKL obtained from three independent experiments. ***, P< 0.001; **, P< 0.01; *, P< 0.05; ns, not significant. [file 13567_2026_1814_MOESM1_ESM.docx]

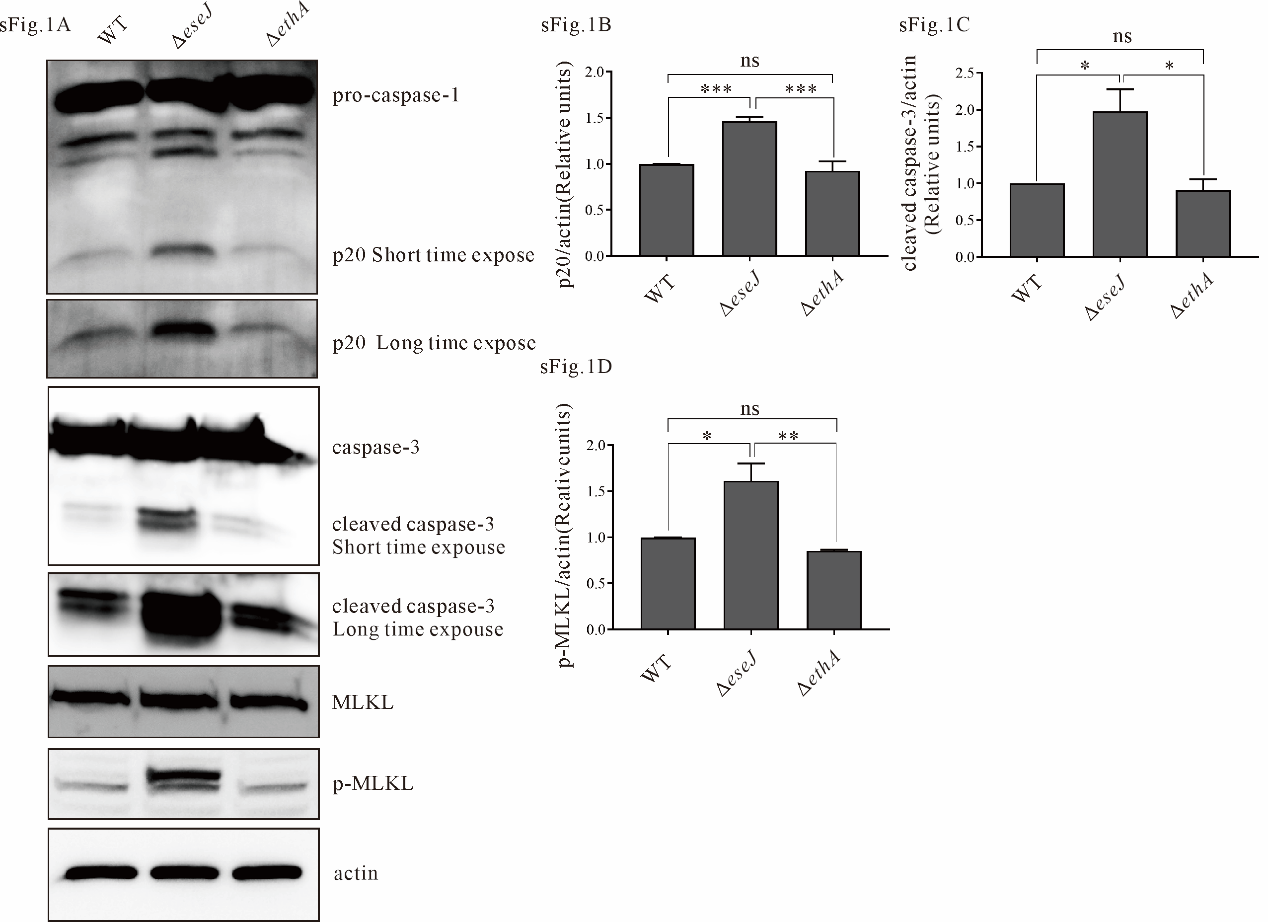


Figure legend:

**sFig.1.** **Depletion of EthA did not affect the levels of PANoptosis stimulated by *E. piscicida* PPD130/91 in J774A.1 macrophages.**

(A) Immunoblotting of cleaved caspase-1 (p20), cleaved caspase-3 and p-MLKL in J774A.1 cells infected with the *E. piscicida* WT, Δ*eseJ* and Δ*ethA* strains. The cell lysates and culture supernatants were precipitated for probing. Actin was used as a loading control to indicate similar amounts of protein loading per lane.

(B, C & D) Quantitative analysis of p20 (B), cleaved caspase-3 (C), and p-MLKL (D) in J774A.1 cells infected with the *E. piscicida* strains shown in sFig. 1A. The levels of p20, cleaved caspase-3 and p-MLKL were quantified using densitometry and normalized to actin levels. The graph shows the relative ratios of p20, cleaved caspase-3, or p-MLKL obtained from three independent experiments. ***, *P* < 0.001; **, *P* < 0.01; *, *P* < 0.05; ns, not significant.
